# A Quantitative Digital Analysis of Tissue Immune Components Reveals an Immunosuppressive and Anergic Immune Response with Relevant Prognostic Significance in Glioblastoma

**DOI:** 10.3390/biomedicines10071753

**Published:** 2022-07-21

**Authors:** Miguel A. Idoate Gastearena, Álvaro López-Janeiro, Arturo Lecumberri Aznarez, Iñigo Arana-Iñiguez, Francisco Guillén-Grima

**Affiliations:** 1 Pathology Department, Clinica Universidad de Navarra and School of Medicine, University of Navarra, 31008 Pamplona, Spain; alopez.44@alumni.unav.es (Á.L.-J.); arturoleaz@gmail.com (A.L.A.); iarana.1991@gmail.com (I.A.-I.); 2 Pathology Department, Virgen Macarena University Hospital and School of Medicine, University of Seville, 41009 Seville, Spain; 3 Department of Preventive Medicine, Clinica Universidad de Navarra, University of Navarra, 31008 Pamplona, Spain; frguillen@unav.es

**Keywords:** glioblastoma, image analysis, immunotherapy, immune barrier, M2 macrophage, microglia

## Abstract

*Objectives*: Immunostimulatory therapies using immune checkpoint blockers show clinical activity in a subset of glioblastoma (GBM) patients. Several inhibitory mechanisms play a relevant role in the immune response to GBM. With the objective of analyzing the tumor immune microenvironment and its clinical significance, we quantified several relevant immune biomarkers. *Design:* We studied 76 primary (non-recurrent) GBMs with sufficient clinical follow-up, including a subgroup of patients treated with a dendritic cell vaccine. The IDH-mutation, EGFR-amplification, and MGMT methylation statuses were determined. Several relevant immune biomarkers, including CD163, CD8, PD1, and PDL1, were quantified in representative selected areas by digital image analysis and semiquantitative evaluation. The percentage of each immune expression was calculated with respect to the total number of tumor cells. *Results:* All GBMs were wild-type IDH, with a subgroup of classical GBMs according to the EGFR amplification (44%). Morphologically, CD163 immunostained microglia and intratumor clusters of macrophages were observed. A significant direct correlation was found between the expression of CD8 and the mechanisms of lymphocyte immunosuppression, in such a way that higher values of CD8 were directly associated with higher values of CD163 (*p* < 0.001), PDL1 (0.026), and PD1 (0.007). In a multivariate analysis, high expressions of CD8+ (HR = 2.05, 95%CI (1.02–4.13), *p* = 0.034) and CD163+ cells (HR 2.50, 95%CI (1.29–4.85), *p* = 0.007), were associated with shorter survival durations. The expression of immune biomarkers was higher in the non-classical (non-EGFR amplified tumors) GBMs. Other relevant prognostic factors were age, receipt of the dendritic cell vaccine, and MGMT methylation status. *Conclusions:* In accordance with the inverse correlation between CD8 and survival and the direct correlation between effector cells and CD163 macrophages and immune-checkpoint expression, we postulate that CD8 infiltration could be placed in a state of anergy or lymphocytic inefficient activity. Furthermore, the significant inverse correlation between CD163 tissue concentration and survival explains the relevance of this type of immune cell when creating a strong immunosuppressive environment. This information may potentially be used to support the selection of patients for immunotherapy.

## 1. Introduction

Glioblastoma (GBM) is the most common primary brain cancer that affects children and adults against which we do not have an effective therapy. Recently, immunotherapy has emerged as an alternative to traditional chemo-radiotherapeutic treatments. It is expected that patients with GBM who have efficient immune responses could be candidates for immunotherapy.

To identify this group of patients, it is necessary to evaluate and quantify key parameters with high precision and reproducibility. Tumor-infiltrating lymphocytes (TILs) have been postulated as relevant biomarkers, as have factors related to the degree of tumor immunogenicity, including the tumor mutational burden, microsatellite instability, and the molecular subtype of GBM [1,2,3].

The antitumor immune response depends on the activation of T lymphocytes by antigen-presenting cells, including macrophages and dendritic cells. The immune response to GBM is the result of a complex balance between inhibition and activation signals exerted on CD8 effector lymphocytes. There are several relevant inhibition mechanisms, including PD-1/PDL1 immune checkpoints, cytotoxic T lymphocyte-associated antigen 4 (CTLA4), FoxP3 regulatory T cells, and immunoregulatory macrophages/plasmacytoid cells [4,5,6,7]. This environment can be modified by treatment with immune checkpoint inhibitors [8]. It is known that programmed cell death protein 1 (PD-1) is an inhibitory receptor induced in activated T cells that regulates CD8 T cell activity in viral infection and cancer. Consequently, it is assumed that PD1 immunostained cells correspond to lymphocytes [9,10].

Tumor-associated macrophage (TAM) infiltration in GBM is thought to arise from microglia and monocytes, which are responsible for one of the more relevant components of inflammation in brain cancer [11]. Resident and infiltrating TAMs are recruited to the growing tumor in response to a variety of tumor-secreted cytokines, including CCL2, GM-CSF, EGF, CXC3CL1, SDF-1, and CSF-1. One important topic that has received increasing attention to date is the role of microglia in GBM. Microglia can be the most important source of macrophages for TAMs in glioblastoma. TAMs show a wide functional spectrum that can be subdivided into two major subtypes. It has been postulated that M1/M2 polarized macrophages play an important role in the microenvironment of GBM [9]. In addition, intratumor macrophages can adopt an M2-like phenotype under the effect of cytokines (e.g., IL-10). In gliomas, the microglia/macrophage M2-like phenotype promotes tumor progression and is involved in immune suppression and angiogenesis, while the M1-like microglia/macrophage phenotype suppresses tumor growth. Several markers have been proposed to identify macrophage polarization phenotypes. The CD163 scavenger receptor is thought to be a selective M2 marker and has been used to assess M2-like macrophage infiltration in various human tumors [12,13,14]. Relatively high levels of CD163-positive macrophages were observed in IDH wild-type GBM without showing a significant difference between the distinct molecular GBM subclasses [15].

We postulated that both the transformation of M2 microglia/macrophages and the accumulation of M2 microglia/macrophage infiltration could be relevant components of immunosuppressive mechanisms in GBM. To evaluate this, we studied immunohistochemically the morphology of immunostained microglia/macrophages, and we quantified the TILs and TAMs in primary human GBMs and analyzed their prognostic impact.

## 2. Methods

### 2.1. Inclusion Criteria

Patients with a primary GBM (patients without clinical or histologic evidence of a less malignant precursor lesion) were resected and received at least a first cycle of adjuvant treatment (radiotherapy plus temozolomide) at the Clinica Universidad de Navarra from January 2009 to December 2015. Patients had not received previous treatment with chemotherapy or radiotherapy. The residual tumor was verified by magnetic resonance imaging. Clinical data were obtained from electronic clinical records. A number of these patients had received the same adjuvant treatment and vaccination with autologous dendritic cells and had been included in a clinical trial (EudraCT: 2009-009879-35 and ClinicalTrials.gov Identifier: NCT01006044 (accessed on 9 July 2022)) [16]. Eligible patients were required to meet the same criteria as the patients not treated by vaccines: aged between 18 and 70 years and with tumors treated by surgery guided by fluorescent microscope.

Data collected included sex, age, Karnofsky performance status (KPS) at diagnosis, tumor location, tumor resection percentage, number of resections, and relapse. Overall survival (OS) data were provided by the neurosurgery and neurooncology departments.

Tumor resection was maximal or near maximal in most cases, with a mean percentage of tumor resection of 98.9%. Out of 76 patients, 26 (34%) relapsed; of the 26 patients who relapsed, 25 (96%) underwent another tumor resection surgery. Of the 76 patients at risk at the beginning of the follow-up, 60 died after a median time at risk of 15.5 months. The shortest survival time after diagnosis was 1.4 months, and the maximum overall survival time was 64.5 months.

### 2.2. Histopathological Study, Digital Image Analysis, and Semiquantitative Evaluation

All tissue samples obtained before the treatment were formalin-fixed and paraffin-embedded. Necrotic areas were disregarded in order to avoid bias in counting. Single marker immunohistochemistry (IHC) was performed on 3–4 µm whole-slide sections representative of the whole tumor. Perivascular lymphocytes and lymphocytes from necrosis-associated areas were excluded from the assessment. The following primary antibodies were used for protein identification: CD163 (clone MRQ-26, Cell Marque, Cal. USA), CD8 (clone C8/144B, DAKO, Glostrup, Denmark), PDL1 (Clone 28-8, DAKO), and PD1 (NAT105, Cell Marque). Human tonsil was used as a positive external control. Staining was carried out using either Ventana Benchmark Ultra Platform (Ventana Medical Systems from Roche Tissue Diagnostics, Arizona, USA) or Autostainer Link 48 from DAKO, following the manufacturers’ instructions. Membranous staining was considered significant, but cytoplasmic and nuclear staining were not.

A *semiquantitative assessment* of the immunostained cells was performed by three blinded observers prior to quantification. The CD8, PDL1, and PD1 cell numbers were evaluated in the hot spot areas. The number of TILs was manually counted by two investigators and an experienced neuropathologist. Scores were re-examined to ensure reproducibility. For CD8 and PD1 infiltration, four scores or categories were defined: 0 for no or very mild presence of CD8 lymphocytes, 1 for lymphocytes frequently encountered along the slide, 2 for dense lymphocyte infiltration, and 3 for dense infiltration with lymphoid aggregate formation. For PDL1, four grades or scores were defined: 0 for no protein expression, 1 for protein expression in isolated cells, 2 for protein expression in cell clusters with incomplete membrane staining, and 3 for complete membrane expression in cell clusters. Excellent intra-observer and inter-observer agreements were reached. In cases of discrepancies in scoring, the mean value was set as the final score. The average of the TIL counts per field for each patient was used for statistical analysis, according to Han et al. [17]. For CD163, a combination of the phenotype and the density of the infiltrate was scored because it made it possible to differentiate between microglial and macrophagic morphology. The morphology is clearly different in both types of cells. Microglia are characterized as cells with long expansions that can be depicted in CD163 immunostaining, whereas macrophages have a rounded cell phenotype without expansions and also use the CD163 molecular marker. Both types of cells, and the intermediate ones, can be scored considering which type of cell is predominant in the tumor. CD163 infiltration was divided into three grades or scores: grade 1 for low or very mild infiltration of macrophages displaying microglial morphology (cytoplasmic expansions), grade 2 for greater infiltration of concurrent microglial and globoid macrophages, and grade 3 for dense infiltration of globoid macrophages grouped in clusters. Areas around necrosis or cell perivascular infiltration were excluded from evaluation.

**Figure 1:**
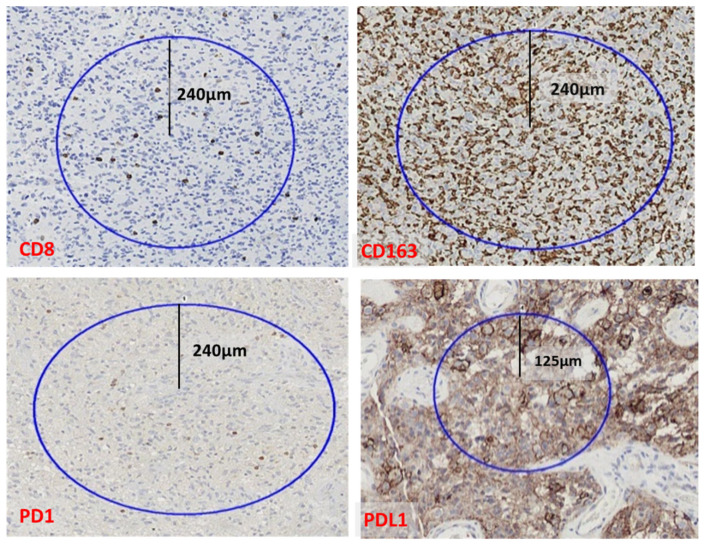
Hot spots, i.e., stained areas with 240 μm radii were considered for CD8, CD163, and PD1, and with 125 μm radii for PDL1. Brown color represents immunostained cells and blue color counterstained nuclei stained with counterstained with Harris hematoxylin.

*Quantification* was assessed by computer-assisted image analysis using the DAKO ACIS III analyzer. Samples that scored 1 or more were further analyzed by image analysis. Slides were scanned using a 10× power scope. The color detection threshold was optimized for the staining technique and applied to all slides. For a quantitative assessment of these preparations, the system requires the areas to be analyzed to be defined as so-called regions of interest (ROIs). The selected areas were defined as the hot spots in the sample for each marker. The percentage of the area occupied by immunostaining and the intensity of the immunostaining were obtained. For CD163, CD8, and PD1, representative circular areas with radii of 240 micrometers (0.18 mm^2^) were analyzed. For PDL1, circular areas with radii of 125 micrometers (0.04 mm^2^) were analyzed. In case of PDL1, a smaller area was utilized because this expression was more focal (more tightly grouped immunostained cells) than the other markers analyzed (Figure 1).

The morphology of the CD163 immunostained cells was not considered in the quantification analysis. Quantitative image analysis was estimated as the ratio of the number of pixels in the brown or blue classes to the total number of pixels in the brown or blue classes to the total number of pixels representing both classes together. Data were expressed, for both, as percent staining. In the same way as in the semiquantitative study, areas around necrosis and cell perivascular infiltration were excluded from quantification. Quantitatively assessed CD163 macrophage infiltration was converted to a categorical variable to perform a concordance test. PDL1 expression in cell types other than tumor cells (macrophages and neurons) was not taken into account because it exhibits slight membranous and cytoplasmic immunostaining and is difficult to measure. In addition, we considered only the clear PDL1 immunostaining in tumor cells, according to Nduom et al. [18].

### 2.3. Molecular Subtyping Approach and MGMT Pyrosequencing

The cases were classified as classical or non-classical GBM according to a simplified approach in such a way that the presence or absence of EGFR amplification was considered a key factor in this distinction [5]. EGFR amplification was studied by silver in situ hybridization (SISH). Probes against the chromosome 7 centromere and EGFR (Ventana Medical Systems) were utilized. SISH was performed on the Benchmark Ultra platform (Ventana Medical Systems), according to the manufacturer’s protocol. Cases were evaluated as positive according to the Colorado score [19] (≥15 signals in ≥10% of the counted nuclei, ratio EGFR/CEP7 ≥ 2, or EGFR gene cluster ≥ 10%).

IDH mutation was evaluated by a combined approach using an immunohistochemical or qPCR by amplification-refractory mutation system (ARMS) approach in 61 tumors (80.2%). First, IDH R132H mutation was assessed by immunohistochemical analysis against IDH1 R132H using clone H09, DIANOVA. Human oligodendroglioma was used as a positive control. A Link 48 Autostainer (DAKO) was used to perform the immunohistochemistry, following the manufacturer’s instructions. The presence of immunostaining in cell cytoplasm was considered a positive result. If cases were negative, they were studied by real-time PCR, Therascreen IDH 1/2 RGQ PCR kit, Qiagen, Hilden, Germany. This kit detects 7 mutations in IDH1 and 5 in IDH2 by analyzing codons 100 and 132 of IDH1 and codon 172 of IDH2.

MGMT methylation status was assessed by pyrosequencing. DNA was obtained from formalin-fixed and paraffin-embedded specimens by an EZ1 Advanced DNA Paraffin Section Card. Pyrosequencing was performed with an MGMT Pyro Kit (Qiagen), and data were analyzed with a PyroMark Q24 System (Qiagen). The established threshold for methylated status was 10%.

### 2.4. Statistical Analysis

The analyses were carried out using the R software package version 4.1.3. (https://www.R-project.org/ (accessed on 9 July 2022)) Descriptive statistics were used using tables and graphs representing the absolute and relative values of the qualitative variables (these data were used to validate the quantitative evaluation and are not the objective of this work), as well as measures of central tendency and variability for the quantitative variables. The normality assumptions for the quantitative variables were verified, where parametric or non-parametric tests were defined. Regarding inferential statistics, bivariate analyses were performed to correlate the percentages of CD163, CD8, PDL1, and PD1, using the Spearman correlation coefficient. The independent sample *t*-test and the Mann–Whitney test were used to compare CD163, CD8, PDL1, and PD1 by molecular subtype. Survival analysis was performed by comparing the curves using the log-rank test (Mantel–Cox) or Gehan–Breslow (generalized Wilcoxon). Cox regression was performed with a non-parametric adjustment of proportional hazards. Statistical significance was established for *p*-values < 0.05.

## 3. Results

### 3.1. Clinical Data

The distribution of clinical features in our sample cohort is presented in Table 1.

Of the 76 patients, 42 were males. The median age was 61, with only four patients younger than 40. All patients received chemoradiotherapy according to standard protocols. The vaccination group also received dendritic cell vaccination as explained in a previous report [14]. These patients were finally included if their residual tumor volume was lower than 1 cc on postoperative radiological examination. The overall median survival in the cohort was 15 months. The median KPS score at diagnosis was 80. Although the KPS scores at diagnosis ranged from 50 to 100, the 25th and 75th percentiles were 80% and 90%, respectively.

### 3.2. Morphological Characterization of the Macrophage Tissue Immune Barrier and Quantification of the CD163 Values

**Figure 2:**
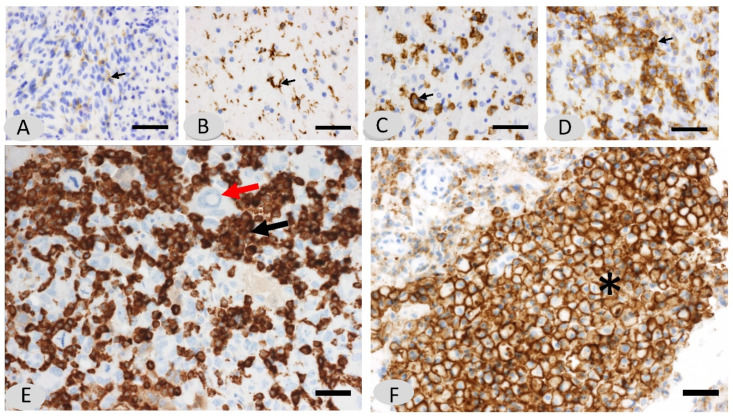
Representation of the gradation of CD163 immunostained cells in GBM, which represents a gradual transition between microglia and macrophages. The different grades of CD163, according to both phenotype and cell density (grades 0 to 3), are shown. The morphological changes clearly support the transformation of microglia into macrophages. The grading shows both the morphology of the immunostained cells and the cell density. (**A**) In grade 0, few quiescent cells corresponding to microglia are observed. Immunostained cells show thin and short expansions. (**B**) In grade 1, the microglia show long and thicker expansions simulating dendritic cells, and the cell density is higher than in grade 0. (**C**) In grade 2, microglia cells are larger and show short expansions. The cell density is higher than in grade 1. (**D**) In grade 3, microglia adopt a round shape without expansions (macrophage phenotype) and form dense aggregates. It can be observed that immunostained cells acquire a round shape and a shortness of dendritic expansions according to a number of immunostained cells. Scale bar: 60 μm (**E**) Note that CD163 immunostained cells form very compact aggregates of macrophages encircling tumor cells. (**F**) A dense aggregate of macrophages is shown in the bottom right of the photograph, forming a so-called “immune barrier” (asterisk). Most of the nuclei counterstained with Harris hematoxylin (blue color) correspond to tumor cells, ×200. Scale bar: 60 μm).

Macrophages and microglia were the predominant populations for every GBM and were randomly distributed throughout the tumor. In general, the grading of immunostaining shows both the morphology of the cell and the cell density because these features are linked. Cells positive for CD163 (an M2-like macrophage) showed three different morphological patterns: a poor cellularity consisting of ramified microglia with slight immunostaining or with a more intense immunopositivity and thicker expansions (grade 1); an intermediate microglia/macrophage aspect of globoid cells with short expansions and a denser cellularity; and a third pattern consisting of a number of dense aggregates of rounded macrophages without expansions (Figure 2). We observed, in CD163 immunostaining with hematoxylin-counterstained slides, that CD163-positive cells formed densely grouped cell areas that apparently do not leave any place for lymphocytes (Figure 2F). In addition, we compared these slides with the corresponding CD8 ones. We called these dense CD163 grouped cells ‘*immune* *barriers*.

The morphological changes clearly support the transformation of microglia to macrophages, which explains the intermediate forms between the two. CD163 immunostaining is an excellent tool for visualizing microglia. In each tumor, a CD163-dominant pattern of infiltration was evident. Interestingly, in a number of cases, the macrophages formed very dense clusters that we called immune barriers or *macrophage immunosuppressive barriers* (Figure 1). These clusters were very compact aggregates of macrophages forming dense nests. We did not identify tumor cells or lymphocytes among these dense macrophage aggregates in the CD163 immunostained slides counterstained with hematoxylin.

**Figure 3:**
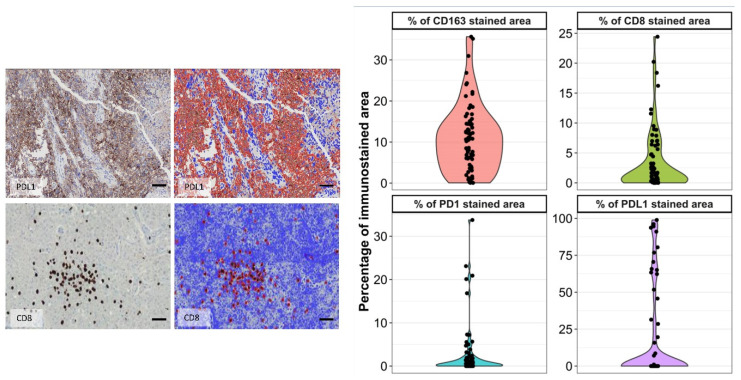
Examples of quantification by computer-assisted image analysis of tumor-infiltrating lymphocytes (TILs). (**Left**) Examples of immunostained PDL1 and CD8 cells (brown color) and how they are recognized by digital analysis (red color). Blue color represents non-selected cells. Scale bar: 60 μm (**Right**) Distribution of values of CD163, CD8, PDL1, and PD1 stained cell image processing.

Seventy-three slides were able to be scanned, digitized, and then quantified with ACIS III (Figure 2). M2-like macrophage infiltration was 10.79% of total cells (sd = 8.18 and range = 0.06–35.64). Infiltration percentage was not normally distributed in our population (Shapiro–Wilk test, *p* < 0.001). An increasing trend in the mean infiltration percentage across grades was observed. The results from the quantitative assessments are summarized in Figure 3.

A reasonably good concordance between semiquantitative and quantitative results was observed for all immune cells (Kappa = 0.54; agreement = 76.71%; *p* < 0.001) (data not shown).

### 3.3. Non-Significant Statistical Differences between Biomarkers in the Vaccination and Non-Vaccination Groups Permits Studying All Cases as a Single Group

When comparing the median values of CD163, CD8, PDL1, and PD1 immune biomarkers from the resection samples obtained before the treatment, it was concluded that no significant differences were observed between immune biomarker values from the vaccinated and unvaccinated groups. This result indicates that the vaccinated and unvaccinated groups of patients presented homogeneity with respect to the parameters. Based on this result, it was decided to take the medians of CD163, CD8, PDL1, and PD1 as cut-off points, using all cases for overall survival correlation regardless of the condition of being vaccinated or not (Table 2).

For the multivariate analysis, different Cox regression models were tested to determine whether there was an interaction between dendritic cell vaccine status and the immune biomarkers in the correlation with the overall survival of the patients and, therefore, whether immune biomarkers were confusing variables. The interactions of %CD163/dendritic cell vaccine and %CD8/dendritic cell vaccine were non-significant (*p*-values 0.828 and 0.968, respectively). Therefore, it can be stated that the final statistical model presented is validated and that there was no interaction between the values of the immune biomarkers and vaccination status in relation to the overall survival of the patients.

### 3.4. CD8, PD1, and PDL1 Characterization and Quantification

**Figure 4:**
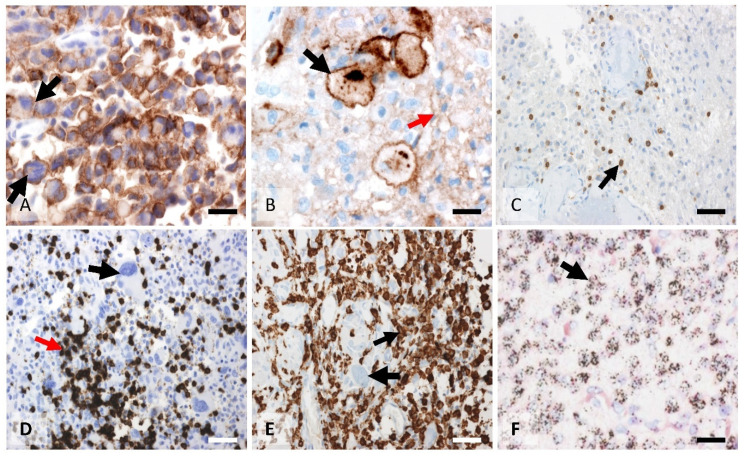
Different representative examples of immunohistochemistry against immune cells. (**A**) PDL1 expression in the tumor cells. Black arrows mark two of these cells (immunostaining against PDL1, ×200. Scale bar: 60 μm). (**B**) Membrane immunostaining with cytoplasmic focal expression against PDL1 in glioblastoma cells (black arrow). Most of the slightly immunostained cells (red arrow) correspond to macrophages (immunostaining against PDL1, ×400. Scale bar: 30 μm). (**C**) PD1 immune expression in lymphocytes (black arrow), immunostaining against PD1, ×200. Scale bar: 60 μm). (**D**) Dense CD8 intratumoral immunostained cells (red arrow). Black arrow shows a tumor cell (immunostaining against CD8, ×200. Scale bar: 60 μm). (**E**) Dense CD163 infiltration corresponding to M2 macrophages (small arrow) and tumor cells (big arrow), (immunostaining against CD163, ×200. Scale bar: 60 μm). (**F**) Amplified EGFR gene (black spots) in tumor cells corresponding to a classical GBM. The black arrow indicates a tumor cell. (Silver in situ hybridization (SISH) against EGFR, ×200. Scale bar: 60 μm).

CD8-positive tumor-infiltrating lymphocytes were frequently encountered in the sample. Only 5% of the samples were devoid of CD8-positive T cells. Immunohistochemistry showed both a perivascular and an infiltrative distribution of T lymphocytes (Figure 4). Perivascular lymphocytic cuffings were observed in 12 out of 76 samples (16%), while most slides showed areas of dispersedly distributed lymphocytes. The mean infiltration found in our samples was 3.34% [sd = 42.5 (0.001–24.42)], (Figure 3).

PDL1 showed a patchy staining pattern with protein expression seen in cell clusters. Great variability in the stained area was found among different patients (standard deviation = 30). Immunostaining was mainly membranous, with a fine and linear pattern (Figure 4). Occasionally, a dot-like, intense cytoplasmic immunostaining was observed. Strong staining was seen in less than 10% of the samples. More than half of the samples were negative for PDL1. A population of cells with poor granular cytoplasmic staining without associated membrane protein expression was seen in 72% of slides. These cells seemed to correspond to macrophages, as was observed in hematoxylin counterstaining and in comparative immunostaining against CD163. However, this conclusion should be confirmed by double immunofluorescence. The mean infiltration found in our samples was 60.74% (sd = 30.1 (7.1–98.9)) (Figure 2). The correlation between different biomarkers is summarized in Table 3.

PD1 expression (Figure 3) was seen in 49 out of 76 samples (64%). PD1 aggregates were seen in less than 20% of samples. The percentage of immunostained areas in hot spots was low, with a mean surface occupied by immunostaining of only 4%. The 76 samples analyzed had a mean immunostained area of 3.3% in hot spots. Mean infiltration found in the cohort was 4.0% [sd = 7.14 (0.04–33.7)], (Figure 2).

### 3.5. CD163 Values Showed a Direct Correlation with Either CD8 or PDL1/ PD1 Infiltration

The percentages of CD163, CD8, PDL1, and PD1 were correlated, with a significant correlation observed between CD163 and CD8 (Rho = 0.563, *p* < 0.001), PD1 (Rho = 0.414; *p* < 0.01), and PDL1 (Rho = 0.544; *p* < 0.05); these were direct linear relationships. In this way, as the CD163 values increased, the CD8, PDL1, and PD1 values increased in parallel. CD8 was related to PD1 (Rho = 0.391; *p* = 0.007) and PDL1 (Rho = 0.511; *p* = 0.026), where the relationship was a linear direct variation; that is, if CD8 increased, PDL1 and PD1 also increased. However, PD1 did not correlate with the expression of PDL1 (Rho = 0.079; *p* > 0.05) (Table 3).

### 3.6. Macrophage Infiltration Correlated with the Non-Classical Subtype of GBM

Forty-four percent of the samples were classical subtypes. Of the cases that were analyzed, no samples were positive for the IDH mutation immunostaining or sequencing that corresponds to primary GBM. Nearly half of the tumors in our sample were classical (44%) with an evident amplified EGFR gene (Figure 3), which is consistent with data published by other authors. Only one sample was unable to be subtyped due to the poor quality of the EGFR result.

**Figure 5:**
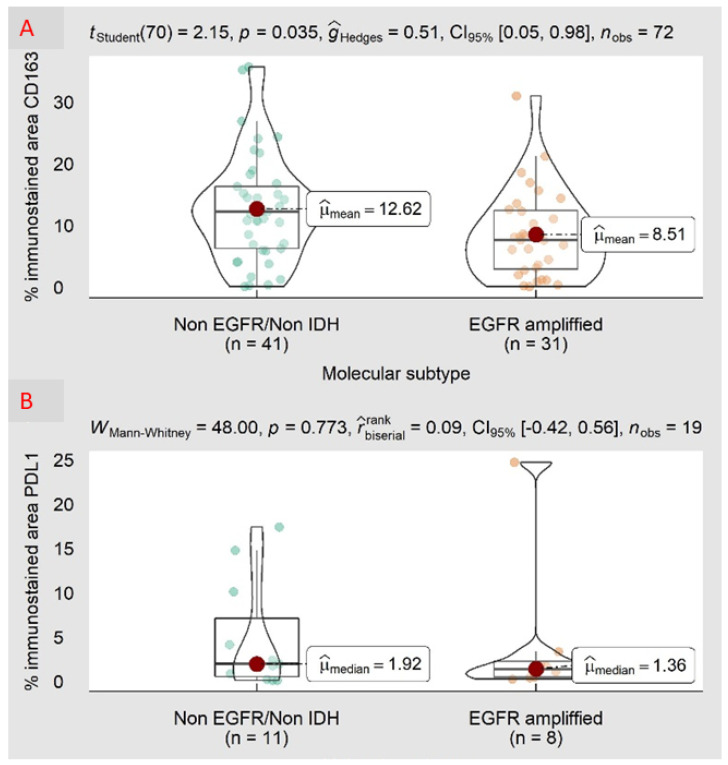

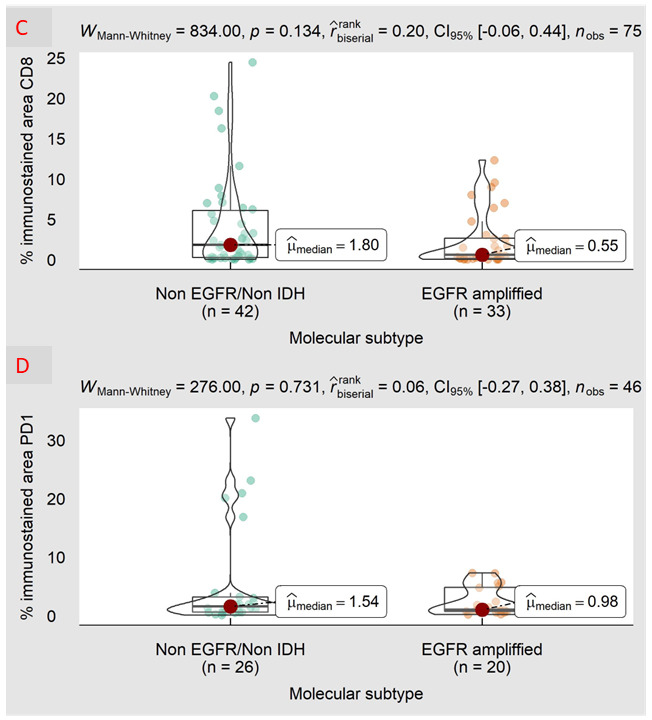
Comparison of the percentages of CD163, CD8, PDL1, and PD1 by molecular subtype (non-EGFR-amplified/non-IDH-mutated versus EGFR-amplified GBM) based on the Student’s *t*-test (**A**) and on the Mann–Whitney test (**B**–**D**). The Student’s *t*-test and Mann–Whitney are presented with their corresponding *p*-values and according to the mean or median values. Graphs are shown with box/violin plots. The percentages of CD163, CD8, PDL1, and PD1 were compared by molecular subtype. It is shown that CD163 exhibited significant differences in the means with a *p*-value of 0.035 and with means of 12.62% for EGFR/non-IDH vs. 8.51% for EGFR-amplified GBM. No significant differences were observed for CD8, PDL1, or PD1.

The mean values of CD8, CD163, PDL1, and PD1 expression in both classical and non-classical GBM are indicated in Figure 5.

The percentages of CD163, CD8, PDL1, and PD1 were compared by molecular subtype. For CD163, significant differences were observed in the means, with a *p*-value of 0.035; the means were 12.62% for EGFR/non-IDH as compared with 8.51% for amplified EGFR. No significant differences were observed for CD8, PDL1, or PD1.

### 3.7. CD163 Macrophage and CD8 Infiltration Correlated with Survival

**Figure 6:**
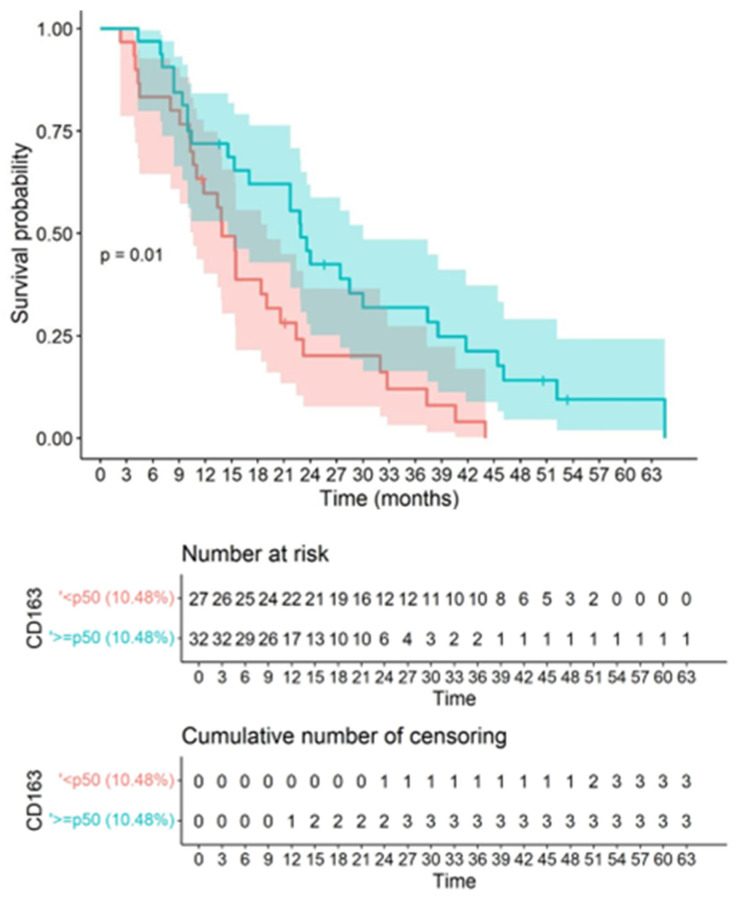
Correlation between CD163 and survival is shown. Kaplan–Meier graph showing overall survival according to the cut-off of quantitatively assessed CD163 immunostaining based on the log-rank test. For the cut-off point of the CD163 percentage, significant differences were observed in the survival curves, with a *p*-value 0.012, where values <p50 (10.48%) presented better survival compared to values ≥p50 (10.48%). The colored bands represent the confidence intervals of the survival curves for each cut-off point.

In the survival analysis, the median value was considered as the cut-off point for CD163, CD8, PDL1, PD1, and age, in order to obtain groups of homogeneous size to relate to mortality. Based on the medians, the following categories were established: CD163: <p50 (10.48%) and ≥p50 (10.48%); CD8: <p50 (1.18%) and ≥p50 (1.18%); PDL1: <p50 (1.79%) and ≥p50 (1.79%); PD1: <p50 (1.31%) and ≥p50 (1.31%); and age: <p50 (age 60) and ≥p50 (age 60). For the cut-off point of the percentage of CD163, significant differences were observed in the survival curves, with a *p*-value of 0.012, where values <p50 (10.48%) presented better survival compared to values ≥ p50 (10.48%). Based on this cut-off point, CD163+ macrophage infiltration showed an inverse correlation with overall survival (OS), supporting the inference that higher CD163 macrophage infiltration is associated with worse outcomes (Figure 6).

### 3.8. CD163 and CD8 Are Independent Biomarkers of Overall Survival and Not Related to Other Relevant Clinical Parameters Such as Age, MGMT Methylation Status, and Dendritic Cell Vaccination

**Figure 7:**
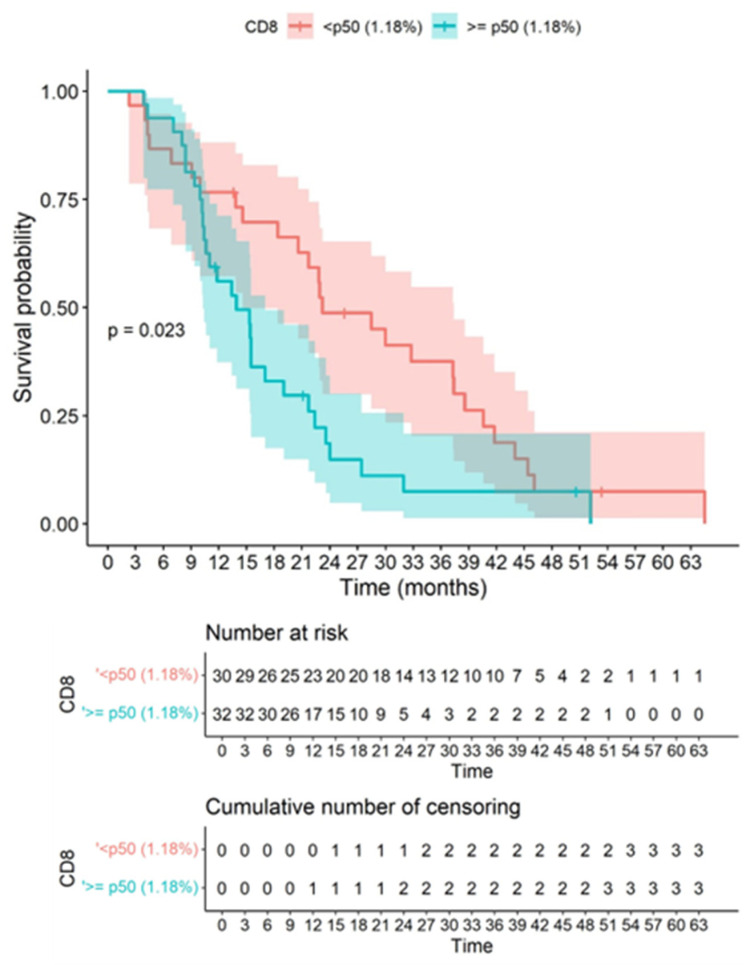
Correlation between CD8 and survival is demonstrated. Kaplan–Meier graph showing overall survival in our cohort according to the cut-point of quantitatively assessed CD8 immunostaining based on the Gehan–Wilcoxon test. At the cut-off point for the CD8 percentage, significant differences were observed in the survival curves, with a *p*-value of 0.023, where values <p50 (1.18%) presented better survival compared to values ≥p50 (1.18%). It is observed that higher CD8 values inversely correlated significantly with a worse overall survival. The colored bands represent confidence intervals of the survival curves for each cut-off point.

This study is the first immunohistochemical analysis of the immune microenvironment in which it is shown that CD8 tissue concentration has an inverse correlation with survival. At the cut-off point for the CD8 percentage, significant differences were observed in the survival curves with a *p*-value of 0.023, where values <p50 (1.18%) presented better survival compared to values ≥p50 (1.18%). Consequently, CD8 infiltration showed an inverse correlation with OS, showing that higher CD8 infiltration is associated with lower OS (Figure 7). PD1 and PDL1 quantitative variables were not associated with survival.

**Figure 8:**
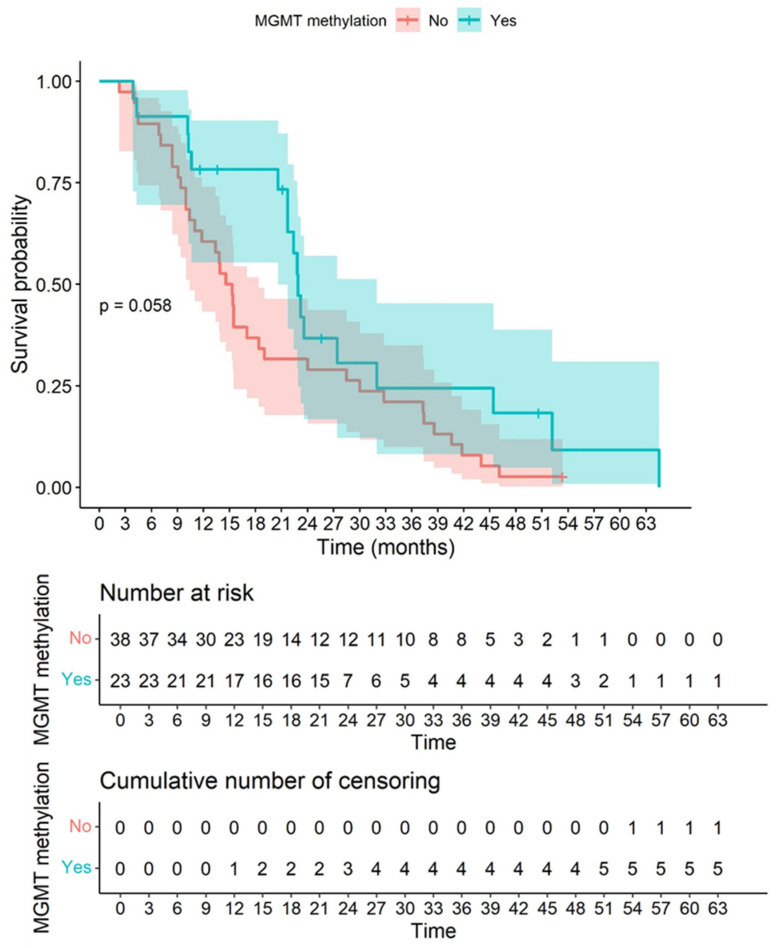
Correlation between MGMT methylation status and survival is shown in this graph. Kaplan–Meier graph showing overall survival in our cohort according to the status of MGMT methylation based on the Gehan–Wilcoxon test. When comparing the survival curves for the presence or absence of MGMT methylation, a *p*-value of 0.058 was observed. Consequently, the presence of methylation presented better survival than non-methylation. The colored bands represent confidence intervals of the survival curves for each cut-off point.

When comparing the survival curves with and without the presence of MGMT methylation status, a *p*-value of 0.058 was observed. In this sense, the presence of methylation was correlated with better survival than non-methylation (Figure 8).

**Figure 9:**
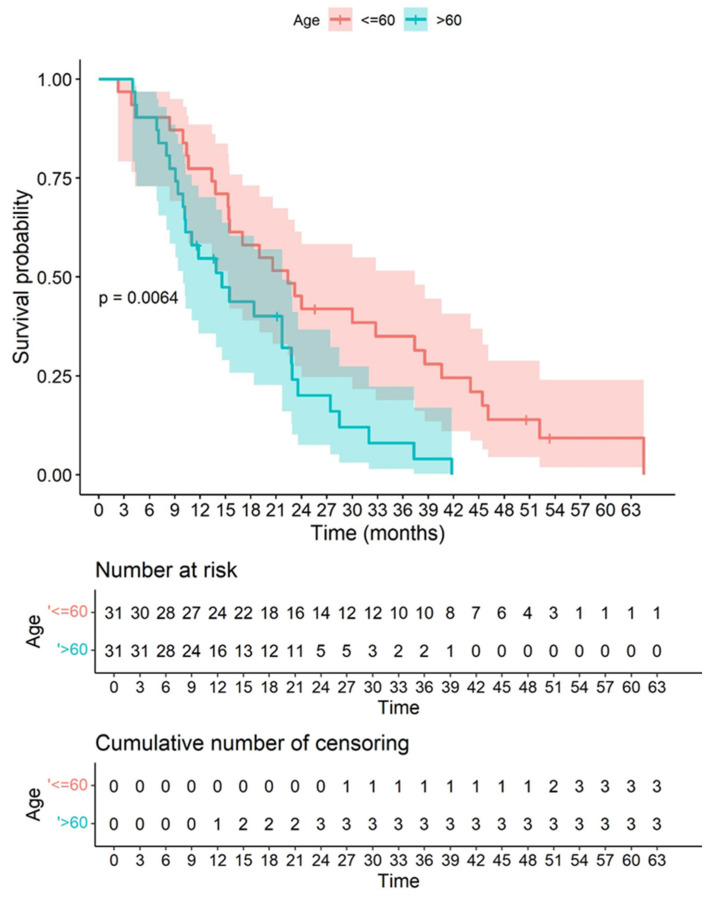
Kaplan–Meier graph showing overall survival in our cohort according to age, with a cut-off of 60 years old based on the log-rank test. By age group, significant differences were observed in the survival curves, with a *p*-value of 0.0064, where patients ≤60 years had better survival than those >60 years. It is observed that being older than 60 years correlated significantly with a worse overall survival. The colored bands represent confidence intervals of the survival curves for each cut-off point.

By age group, significant differences were observed in the survival curves, with a *p*-value of 0.0064, where patients ≤60 years had better survival than those >60 years (Figure 9).

Finally, when comparing the survival curves by molecular subtype, no significant differences were observed.

**Figure 10:**
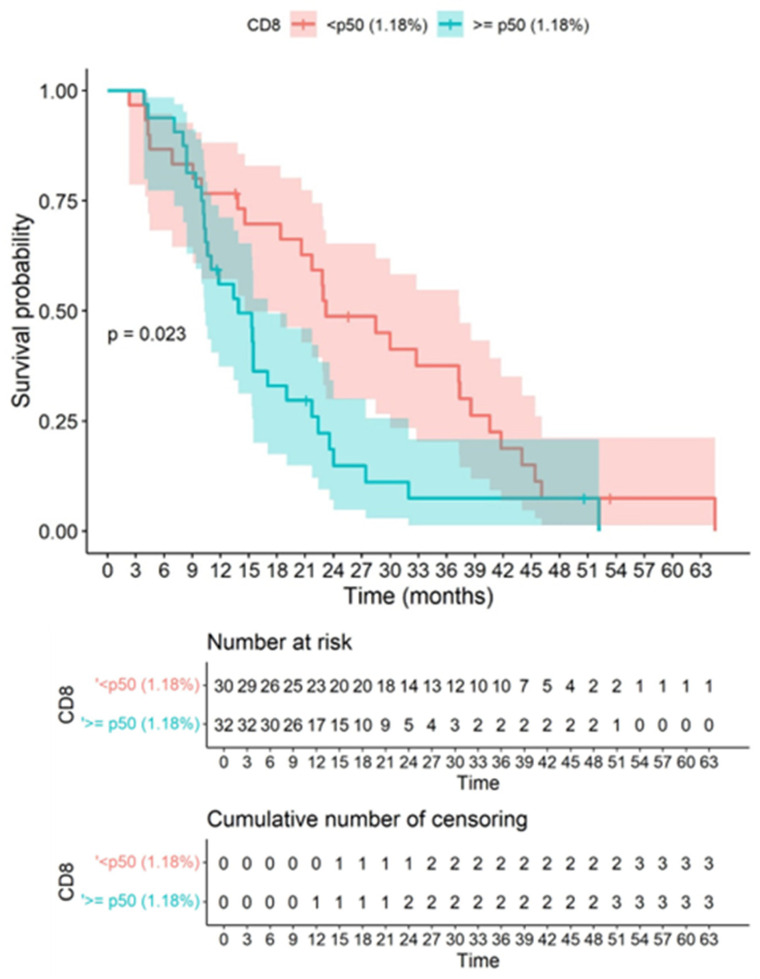
A significant correlation between dendritic cell vaccination and survival was found. Kaplan–Meier graph showing overall survival in our cohort according to dendritic cell vaccination status based on the Gehan–Wilcoxon test. It is observed that vaccination correlated significantly with a better overall survival. Survival curves by dendritic cell vaccination status were compared, and significant differences were observed, with a *p*-value of 0.01, where vaccination presented better survival compared to no vaccination. The colored bands represent confidence intervals of the survival curves for each cut-off point.

Survival curves by dendritic cell vaccination status were compared; significant differences were observed, with a *p*-value of 0.01, where vaccination correlated with better survival than non-vaccination (Figure 10).

According to the results, it is possible to postulate that the effect of vaccination on survival is non-dependent on biomarker values.

Univariate and multivariate Cox regressions were also performed to analyze the impact of immune markers on OS (Table 4).

Other variables that had an impact on OS (age, vaccination, and MGMT methylation status) were included in the multivariate model. Given the homogeneity of KPS scores at diagnosis and tumor resection during surgery found in our sample, we decided not to include these two variables in the model. In the univariate analysis, a significant correlation (*p*-value < 0.05) was observed for %CD163, %CD8, dendritic cell vaccination, and age. Values of %CD163 ≥ p50 (10.48%) represented a 2.04 times higher probability of not surviving. Values of %CD8 ≥ p CD8 ≥ p50 (1.18%) represented a 1.87 times higher probability of not surviving. Cell vaccination was presented as a protective factor against mortality, while patients aged > p50 (60 years) had a 2.18 times higher probability of not surviving. On the other hand, MGMT methylation status showed a *p*-value of 0.062.

We conducted a multivariable Cox regression model introducing the possibly significant variables that were associated with poor survival in the univariate analysis, including MGMT methylation status (Table 4). The Cox model presented a 74% concordance, and the likelihood ratio, Wald, and Score (log-rank) tests were significant, indicating that the model is correct. The variables included in the model fulfilled the assumption of the proportionality of risks. Significance (*p*-value < 0.05) was observed for %CD163, %CD8, MGMT methylation, dendritic cell vaccination, and age (Table 4). Values of %CD163 ≥ p50 (10.48) indicated a 2.50 (1.29–4.85) times higher probability of not surviving. Values of %CD8 ≥ p50 (1.18) indicated a 2.05 (1.02–4.13) times higher probability of not surviving. Age > p50 (60 years) represented a 2.74 (1.48–5.07) times higher probability of not surviving. An MGMT methylation value of 0.39 (0.19–0.79) and a dendritic cell vaccination value of 0.50 (0.26–0.94) were found to be significant protective factors. In summary, it can be assumed that %CD163 and %CD8 are prognostically independent immune biomarkers for GBM because they were not associated with other significant prognostically relevant clinical or molecular parameters.

## 4. Discussion

### 4.1. Automated Digital Image Analysis Is a Relevant Tool in the Objective Quantification of TILs and Reduces the Inter- and Intra-Observer Variability Seen in the Conventional Semiquantitative Approach

The immune response to GBM is the result of a series of activation and inhibition mechanisms. For some time now, the use of digital image analysis has been becoming more popular. For instance, it has been used to analyze macrophages in gastric and breast cancers [20]. Image analysis faces challenging obstacles, such as standardizing image acquisition and image format and managing color [21,22]. However, digital image analysis offers new possibilities in the field of pathology. The potential for obtaining numerical data and the ability to distinguish between slightly different values make it a promising tool in the search for tissue biomarkers [23]. Image analysis may come to be accepted as a useful technique in clinical practice; however, validation processes must be carried out before its introduction. In particular, digital image analysis may help GBM patients by creating a prognostic immunoscore that could integrate the most relevant immune parameters. In the work reported here, we explore image analysis as a method to quantify immune-related parameters. Only a few isolated publications have reported the use of image analysis to quantify TILs in GBM [24,25]. Our work is a study of the quantification of the immune landscape based on digital image analysis, with validation by a prior semiquantitative analysis. In addition, a clinical correlation of immune parameters in a large series of GBM patients was available.

One of the advantages of computer-assisted image analysis over traditional semiquantitative assessment is the reproducibility of results; results are not dependent on the personal experience of the observer in the evaluation of particular cell density. The ability to measure immune markers as continuous variables increases the chances of detecting slight differences between patients that are obscured when gross semiquantitative assessment is performed. In addition, as we have shown in the comparison between immunostained and digitalized images in an optimized digital setting, the identification of immune expression is of high quality. Overall, we strongly recommend the use of computer-assisted image analysis in future studies as a useful method of objectifying the expression of immune markers. It is anticipated that image analysis will become more important in this field, with improvements in the reliability and capacities of new image analyzers.

Of course, double immunostaining against CD8 and PDL-1 would have been interesting to explore concurrently with several biomarkers expressed in the tumor. However, although it was not the objective of this work, we indirectly studied the relationship of both parameters, and it was concluded that a higher CD8 expression was correlated with a higher expression of PDL-1. In addition, we found a strong concordance between manual cell counting and the digital analysis of immunohistochemical specimens. In summary, digital analysis is an innovative approach allowing a valuable, accurate, and more practical approach than manual cell counting.

### 4.2. CD163-Positive Macrophage Infiltration in the Tumor Forms Dense Immune Clusters among the Tumor and Effector Cells

The intratumor macrophages can adopt an M2 phenotype in response to certain cytokines. Molecules such as CD163 or arginase have been proposed in the past as surrogate markers of the M2 phenotype [26,27,28,29]. M2 macrophages represent a critical component of the immune suppressive niche described in cancer. We have shown intermediate phenotypes in between microglia and macrophages in the immunostaining against CD163, in accordance with the known cell transformation potential of microglia [30]. In cases with high numbers of M2 macrophages, dense intratumor macrophage infiltration was seen that apparently was more cellular than the tumoral cells themselves. It was demonstrated that macrophages could form dense or cohesive aggregates of CD163 cells among the tumor cells, which we called the *“immune barrier”*. Moreover, it can be postulated that dense macrophage infiltration could physically separate tumor cells from the effector CD8 lymphocytes. We did not find lymphocytes among the macrophage aggregates in the CD163 immunostained slides counterstained with hematoxylin. Although no coimmunostaining was performed, the morphological observations based on CD163 immunostaining counterstained with hematoxylin showed that CD163 immunostained cells occupied tumor areas as densely grouped cells that did not leave any place for lymphocytes. In these areas, it is possible to check whether CD8 cells are placed inside the CD163 grouped cells or not. This is the reason why we postulate that macrophages form immune barriers in the sense of both “physical” and functional units. We are developing this idea using coimmunostaining to prove this hypothesis.

In this work, it was observed that brain macrophages associated with glioma seem to originate from tissue-resident microglia because a progressive morphologic transformation from microglia to macrophage has been observed in the tumors. Furthermore, a transformation from monocytes to macrophages has been described experimentally [11,31]. CD163 immunostaining is an excellent tool to visualize the microglia phenotype as it has been reported [32]. In fact, the authors of this work hypothesize that CD163-positive microglia are activated cells based on their morphology. We did not focus our study on the origin of TAMs. However, we postulate that the majority of the TAMs represent activated brain-intrinsic microglia that create a supportive stroma for neoplastic cell expansion and invasion. Several studies have argued that the expansion of microglia during microgliosis (microglial activation) results mainly from the local expansion of existing resident microglia. Experimentally, when two models of acute and chronic microglia activation were used, no microglia recruitment from the blood circulation was found [33]. In addition, it was postulated that resident microglia, and not peripheral macrophages, are the main source of brain tumor mononuclear cells [34]. We think that the evident morphological transformation of microglia into macrophages supports our hypothesis that TAMs in glioblastoma originate at least partially from tissue-resident microglia.

The mechanisms by which GBM cells recruit and modulate TAM functions/phenotype are somewhat complex. The activation of TAMs is a dynamic process, transitioning between a proinflammatory phenotype (characterized by inflammatory antitumor responses) and an anti-inflammatory and immunosuppressive phenotype. Tumor cells release a variety of chemokines, cytokines, and growth factors that subsequently lead to the recruitment of TAMs, including monocyte chemoattractant protein-2 (CCL2), which is a critical chemokine for TAM activation. [35]. In this way, TAMs account for one important part of the GBM bulk. TAMs can adopt both M1 and M2 phenotypes in the setting of GBM, but there are a variety of genetic and epigenetic factors that induce the polarization of TAMs toward a stable pro-tumor M2-like phenotype in a hypoxic setting [36]. Activated microglia release interleukin IL-6 and relevant growth factors, including epidermal growth factor (EGF), vascular endothelial growth factor (VEGF), and transforming growth factor-β (TGF-β), which stimulate glioblastoma cell invasion and angiogenesis [37]. The expression of arginase 1 has been proposed as a relevant factor, and arginine deprivation alters microglial polarity to a proinflammatory state [38]. Thus, the interrelation of TAMs and tumor cells is one of the most interesting and complex phenomena in the development of GBM.

### 4.3. Quantified CD163-Positive Macrophage Infiltration Could Be Postulated as a Significant Independent Biomarker of Survival

M2 Macrophages are a heterogeneous population of myeloid cells with an immune suppressive phenotype. Based on our results, we believe that macrophage cell infiltration could be a relevant immunosuppressive mechanism in GBM. The immunosuppressive effect of CD163-positive macrophage infiltration can be deduced from the fact that macrophage infiltration was associated with reduced survival. Moreover, it has been reported that M2 macrophages may block the immune system by a close interrelationship with the effector cells through the so-called immune synapses [39]. These results agree with the postulated role of M2 macrophages in GBM [40]. We have observed that a high expression of M2 macrophages is an independent prognostic factor, as has been postulated by other authors. Sørensen et al. [41] performed automated quantitative evaluation of M2 macrophages using a different marker (CD204) and found that M2 macrophages held an unfavorable prognostic value in high-grade gliomas. Other authors [42] reported a significant correlation between CD163 expression and survival, although the study in question was not based on an immunohistochemical evaluation. In another report using automated image analysis, a significant correlation was observed between CD163 and survival, but not in a Cox regression model [25]. On the other hand, in a recent study using a digital analysis approach, CD163 expression had no significant prognostic significance [15]. In summary, despite these contradictory results in the literature, we strongly support the immunosuppressive effect of CD163 as an expression of an M2-like effect. Interestingly, our results can explain why most clinical trials involving immune checkpoint inhibitors have not proven clinically effective.

From the therapeutic point of view, theoretically, it would be possible to eliminate this postulated CD163 immunosuppressive barrier by means of antagonists of myeloid cells/immunosuppressive macrophages. The pernicious effect of M2 macrophage infiltration opens the door to new targeted therapies based on avoiding this phenotype or blocking its effector functions [43]. CSF-1R inhibition has been used for this purpose in murine GBM models, showing good results [44]. Furthermore, we have reported that no significant changes were seen in parameters such as the activity of T cells, PD-L1 expression, or myeloid/phagocytic cell markers in a nivolumab phase II clinical trial in patients with GBM treated with nivolumab through a comprehensive immunobiological comparative study between pre- and post-treatment tumor biopsies in respect to controls [8].

### 4.4. The Beneficial Effect of Vaccination on Survival despite a Strongly Immunosuppressive Microenvironment

We are aware that a group of patients was treated with vaccines and that an interaction between vaccination and immune biomarkers could be considered. However, when comparing the median values of the immune biomarkers from the resection samples, it was concluded that the vaccinated and unvaccinated groups of patients presented homogeneity with respect to the parameters. Furthermore, by testing different Cox regression models, it was demonstrated that an interaction between the dendritic cell vaccine status and the immune biomarkers in correlation with overall survival did not exist. Consequently, all the immune biomarker data could be considered for overall survival correlation regardless of the condition of being vaccinated or not. It should be noted that, in our series, classical prognostic parameters for GBM, such as age and MGMT status, correlated with survival; this fact supports the conclusion that it was a representative sample of this type of neoplasia.

According to the previous explanation, it is possible to postulate that the effect of vaccination on survival was non-dependent on the biomarker values. Although a beneficial effect of the vaccines was produced, we postulate that vaccination was unable to induce a clinically more relevant response, most probably because of the presence of the strongly suppressive microenvironment. This immune response correlates with the significant differences observed between proliferation in samples taken before vaccine administration and samples obtained from patients after they received vaccines [16]. Only in 15 vaccinated patients were we able to compare CD8 infiltrate in the primary tumor vs. the recurrence in the homogeneous conventionally treated non-vaccinated group. It could be concluded that an increase in CD8 cells in the recurrence was observed only in the vaccinated group compared to the corresponding pre-treated samples (vaccine group: pretreatment (mean and standard deviation values) −1.73 (0.79), post-treatment −2.06 (0.59); *p* = 0.054; control group: pre-treatment −0.86 (0.51); post-treatment −1.26 (0.7); *p* = 0.173) (data included as Supplementary Table S1).

### 4.5. Higher CD8 Intratumor Infiltration Is Associated with a Hostile Microenvironment Created by the Tumor and Is a Significant Independent Prognostic Biomarker That Correlates with Shorter Survival

The findings in the correlation analysis of this study are novel. In this work, we found a direct association between CD8 infiltrating lymphocytes and both the PDL1–PD1 axis and CD163 expression. Although CD8 activity was not specifically studied in this work, a conclusion about the functionality of these cells can be deduced from the significant correlation of these effector cells with both overall survival (inverse correlation) and the immunosuppressive tumor component. The association of CD8 with poor survival is surprising compared with other studies in the literature that addressed this aspect previously. Tumors that have a higher density of CD8 infiltration were found to express higher levels of CD163, PDL1, and PD1. This correlation has been observed by other authors, but those studies were not based on a digitalized immunohistochemical evaluation [42]. Thus, the significant correlations between the values of PD1 and CD8 infiltration are consistent with the fact that PD1 is a cell surface receptor expressed by CD8-positive lymphocytes. It is critically important to understand why anti-PD-1 does not work in GBM patients and whether it will require other sorts of immunomodulatory agents or immune checkpoint blockers to energize the T cells. The tumoral immunosuppressive effect may cause the exhaustion of the cytotoxic T cell response. The explanation for this apparently surprising association can be found in the fact that, although CD8 effector cells are present in the tumor, these cells are anergic, as postulated by other authors [45]. This anergy may be related to the mechanisms of inhibition discussed above, i.e., the immunosuppressive M2 macrophages and PD1–PDL1 checkpoint inhibitor expression. Controversy exists about this point because, in one study, high CD8 infiltration was associated with long-term survival [46,47,48]. On the other hand, in several reports, CD8 T cell infiltration was not associated with longer survival in GBM [17,25]. Finally, other authors found that lymphocyte infiltration was associated with a worse prognosis [39]. We think that this discrepancy can be attributed to a simple methodological difference. This divergence may be related to the use of quantitative methodology to determine the extent of the immune infiltration. In summary, we conclude that CD8 infiltration is an independent predictor of survival because the more CD8 cells there are in the tumor, the stronger the anergic effect of the hostile microenvironment is on the antitumoral immune response.

Although the functional state of the CD8 effector cells has not been expressly studied, it can be postulated that the inverse relationship between CD8 cell tissue concentration and survival may reflect an inefficient or blocked cell and a higher immunosuppressive tumor environment. In other words, the greater the number of effector cells, the greater the immunosuppression and the biological aggressiveness of GBM. We think that the inefficiency of CD8 is based on three arguments: first, the inverse relationship between CD8 cell tissue concentration and survival may reflect an inefficient or blocked cell; secondly, the strong correlation between CD8 and immunosuppressive mechanisms in the sense of higher CD8 density supports the argument that CD8 cells could be blocked by CD163 positive macrophages and the PD1/PDL1 axis; and thirdly, according to the literature, it has been reported that T cell exhaustion is a major contributor to the failure of T cells in GBM [49].

In our opinion, the existence of cohesive aggregates of CD163 cells in GBM has not been emphasized in the literature. We observed dense aggregates of CD163 immunostained macrophages intermixed with tumor cells. The cases with dense macrophagic aggregates corresponded with higher numbers of quantified CD163 immunostained cells, and both correlated directly with worse prognosis.

Our idea is that both the transformation of M2 microglia/macrophages and the accumulation of M2 microglia/macrophage infiltrate could be relevant components of the immunosuppressive mechanisms in GBM. In our study, we compared two relevant immunosuppressive mechanisms, and we concluded that microglia have significantly higher relevance in relation to overall survival than PD1/PDL1 axis expression in tumor cells. We postulate a new concept tat we call the “immune barrier”, which includes two aspects: firstly, macrophages can develop cell clusters to impede interaction between CD8 cells and tumor cells; secondly, macrophages impede the movement of CD8 cells, as reported by Peranzoni et al. [50].

With respect to PDL1/PD1 immunohistochemical expression in GBM, contradictory results have been published about the prognostic significance of the expression of PDL1 in GBM [7,18,51]. In a recent systematic review and meta-analysis [52], it was concluded that high expression of PDL1 in GBM was associated with poor survival of patients, but only in Asian patients with PD-L1-positive expression (CPS ≥ 1). Our work confirms that the quantitative determination of PDL1 expression is not a prognostic factor in European patients.

### 4.6. Microglia/Macrophage Infiltration Is Higher in the Non-Classical Subtype of GBM

Recently, it has become accepted that there are four molecular subtypes of GBM, known as classical, mesenchymal, proneural, and neural [53]. It is known that the mesenchymal subtype of GBM has higher levels of TILs than other subtypes [2,54,55]. Our objective was to compare the classical subtype to the other subtypes, among which mesenchymal is the most frequent. For this objective, EGFR amplification is an excellent approach. The presence of this molecular alteration is sufficient to typify a GBM as a classical subtype. In fact, EGFR amplification was observed in 97% of the ‘classical’ subtype samples [19,56]. As observed in the present work, the non-classical subtypes of GBM show higher infiltration of CD163-positive macrophages than the classical subtype, but not with respect to effector cells or the immunosuppressive PDL1–PD1 axis. These results agree with a recent report, but we considered this parameter as the number of cells per area [25]. Some authors have explained the lower levels of immune cellularity in the classical subtype by EGFR amplification through the attenuation of MHCI and MHCII expression [57].

Finally, we propose a combination of molecular parameters and quantified immunohistochemical markers that could be used as a feasible approach to profile the tumor immune microenvironment. How can the identified surrogate markers be used for the selection of patients for immunotherapy? Theoretically, patients with both high CD8 and low CD163 values could be good candidates.

With respect to possible selection bias in the study, we accept that it may exist even in a randomized clinical trial when the participants do not come from the target population. Although selection bias cannot be ruled out, it was diminished in this study because patients represented the target population to which the study results will be applied. Furthermore, the counting of the immune infiltrate was carried out in unvaccinated and vaccinated groups before their respective treatments. Moreover, comparisons for each of the immune parameters showed no statistically significant differences in both groups. Another issue was that the selected assessment areas on pathological slides are subject to inherent bias. To optimize the digital pathology procedure, we carried out a comparison of immunohistochemical intensity data derived using pixel analysis software versus pathologist visual scoring, obtaining a high correlation (data not shown), according to Rizzardi et al. [58]. In order to support the reproducibility of our experiments, we used antibodies routinely used in the clinical setting. In addition, the selected areas were defined as representative ROIs of the tumor that the algorithm analyzes to yield a score that is intended to represent the whole tumor. These results need to be replicated in additional studies.

In conclusion, we propose the use of digital image analysis to quantify TILs in order to obtain more precise values for clinical–pathological correlations. In this way, it can be shown that the expression of CD163 (M2 macrophage) in GBM is significantly associated with a poorer prognosis. In addition, the higher values of CD8 lymphocytes in GBM are also associated with a poorer prognosis, which could be explained as a reflection of an anergic state of the effector cells. We hypothesize that M2 macrophage recruitment and PD1–PDL1 expression may be a defensive strategy upregulated by GBM in response to TILs. This hostile microenvironment is a more important factor in the non-classical subtypes of GBM.

## Figures and Tables

**Table 1:** Relevant clinical data for the GBM series.

Parameters	Data
Males (*n*, %)	42 (55)
Age (years) (median, p25–75)	61 (52–68)
Dendritic cell vaccination (*n*, %)	33 (42.1)
Overall survival (months) (median, p25–75)	15.45 (9.7–27.9)
Karnofsky at diagnosis (median, p25–75)	80 (80–90)
Percentage of tumor resection (mean range)	98.9 (90.5–100)
Relapse (*n*, %)	26 (34)
>1 resection	25 (33)

**Table 2:** Comparison of the percentages of CD163, CD8, PDL1, and PD1 by dendritic cell vaccination status.

Parameters.	Dendritic Cell Vaccination		*p*-Value
	No	Yes	
%CD163 (mean) (sd) ^1^	11.59 (9.26)	9.64 (6.30)	0.144
%CD8 (median) (IQR) ^2^	7.25 (1.95–11.06)	4.72 (0.79–8.46)	0.494
%PDL1 (median) (IQR) ^2^	7.1 (0.62–16.73)	1.36 (0.39–1.83)	0.061
%PD1 (median) (IQR) ^2^	2.12 (1.5–4.51)	1.53 (0.3–19.12)	0.460

Legend: sd = standard deviation, IQR = interquartile range; ^1^ = *t*-test, ^2^ = Mann–Whitney test.

**Table 3:** Correlation between the biomarkers CD163, CD8, PDL1, and PD1 (%) in glioblastoma.

Variables	%CD163	%CD8	%PDL1	%PD1
%CD163	1	0.563 *	0.544 *	0.414 *
		*p* (<0.001)	*p* (0.016)	*p* (0.005)
%CD8		1	0.511 *	0.391 *
			*p* (0.026)	*p* (0.007)
%PDL1			1	0.079
				*p* (0.770)
%PD1				1

Note: Spearman (Rho) correlation, * significant.

**Table 4:** Cox regression model, univariable and multivariable, of immune markers and other clinically relevant molecular parameters.

Parameter	Hazard Ratio	95%CI	*p*-Value
***Univariable model***			
%CD163 ≥ p50 (10.48%)	2.04	1.15–3.61	0.014 *
%CD8 ≥ p50 (1.18%)	1.87	1.08–3.24	0.025 *
%PDL1 ≥ p50 (1.79%)	1.55	0.57–4.23	0.392
%PD1 ≥ p50 (1.31%)	1.12	0.57–2.22	0.738
MGMT methylation	0.57	0.32–1.03	0.062
Dendritic cell vaccine	0.49	0.28–0.85	0.012 *
EGFR (not classical)	1.41	0.83–2.42	0.207
Tumor resection	0.88	0.77–1.03	0.111
KPS	1.00	0.98–1.03	0.900
Age > p50 (60 years old)	2.18	1.23–3.88	0.008 *
***Multivariable model***			
%CD163 ≥ p50 (10.48%)	2.50	1.29–4.85	0.007 *
%CD8 ≥ p50 (1.18%)	2.05	1.02–4.13	0.045 *
MGMT methylation	0.39	0.19–0.79	0.009 *
Dendritic cell vaccine	0.50	0.26–0.94	0.032 *
Age > p50 (60 years old)	2.74	1.48–5.07	0.001 *
*Model validation*			
Concordance = 0.74 (SE = 0.035)			
Likelihood ratio test			<0.001 **
Wald test			<0.001 **
Score (log-rank) test			<0.001 **
*Proportional hazards validation*			
%CD163 ≥ p50 (10.48%)			0.85 ***
%CD8 ≥ p50 (1.18%)			0.22 ***
MGMT methylation			0.60 ***
Dendritic cell vaccine			0.40 ***
Age > p50 (60 years old)			0.37 ***
Global			0.67 ***

Note: * Significant variable; ** very significant variable; *** meets proportional hazards assumption. Data between parentheses correspond to median values.

## Data Availability

The datasets used and/or analyzed during the current study are available from the corresponding author on reasonable request.

## Supplementary Materials

The following supporting information can be downloaded at: https://www.mdpi.com/article/10.3390/biomedicines10071753/s1, Table S1: Comparative table of Tumor Infiltrating Lymphocytes (TILs) in the vaccinated group versus the control group in the primary tumor and in post-treatment recurrence.
