# Supplementary material for: A Quantitative Digital Analysis of Tissue Immune Components Reveals an Immunosuppressive and Anergic Immune Response with Relevant Prognostic Significance in Glioblastoma
Source: Biomedicines. 2022 Jul 21;10(7):1753. doi: 10.3390/biomedicines10071753 (PMC9313250; doi:10.3390/biomedicines10071753)
Supplement: Supplementary file 1 [file biomedicines-10-01753-s001.zip › biomedicines-1753295-supplementary.pdf]

Supplementary table

TABLE S1: Comparative table of Tumor Infiltrating Lymphocytes (TILs) in the vaccinated group versus the control group in the primary tumor and in post-treatment recurrence.

Groups	Samples	TILs		
		CD3*	CD4*	CD8*
Vaccine	Primary Tumor	1.13 (0.74)	0.73 (0.59)	0.86 (0.51)
	Recurrence	1.4 (0.91)	0.86 (0.64)	1.26 (0.7)
		p=0.217	p=0.499	p=0.054
Control	Primary Tumor	1.93 (0.79)	1.33 (0.81)	1.73 (0.79)
	Recurrence	2.06 (0.59)	1.2 (0.56)	2.06 (0.59)
		p=0.546	p=0.433	p=0.173

(*) Mean and standard deviation values.
